# A Case Report on Successful Resuscitation of a Two-Month-Old Infant in the Emergency Room: Neonatal Resuscitation Program (NRP) Guidelines in Practice

**DOI:** 10.7759/cureus.38291

**Published:** 2023-04-29

**Authors:** Joshua A Jogie

**Affiliations:** 1 Faculty of Medical Sciences, The University of the West Indies, St. Augustine, TTO

**Keywords:** infant respiratory distress, emergency room resuscitation, neonatal resuscitation program (nrp), nrp guidelines adherence, chest compressions

## Abstract

Infants that appear with respiratory distress or cardiac arrest require immediate attention, and neonatal resuscitation is a crucial skill that can significantly impact the outcome. Here, we discuss a case of a two-month-old baby who needed ER resuscitation. The patient needed immediate assistance due to respiratory distress and cyanosis. This case study emphasizes how crucial it is to follow the Neonatal Resuscitation Program (NRP) algorithm because it allows the patient to have a successful outcome. Regarding the decision on whether to use NRP or Pediatric Advanced Life Support (PALS) guidelines for the two-month-old infant, it was ultimately decided to use the NRP guidelines. This decision was based on the preference of the institution. This case was successfully handled, highlighting the importance of complete training and adherence to the NRP recommendations for healthcare workers involved in neonatal care.

## Introduction

When an infant experiences cardiac arrest or respiratory distress, neonatal resuscitation is a necessary life-saving technique. To standardize the approach to neonatal resuscitation and produce better results, the Neonatal Resuscitation Program (NRP) guidelines were published [1]. This case report describes the successful resuscitation of a two-month-old baby in the ER, emphasizing the need to follow the NRP recommendations.

## Case presentation

The parents of a two-month-old male newborn took him to the ER when he suddenly developed cyanosis and had trouble breathing. The infant was previously healthy and had no serious medical history. When he arrived, the child was cyanotic, moaning, and flaring his nose, suggesting respiratory distress. The baby's oxygen saturation (SpO2) was 70%, and his heart rate was 60 beats per minute. It was impossible to get the baby's blood pressure.

Immediate management: application of NRP guidelines

The NRP recommendations state that the first actions in newborn resuscitation should include warming the infant, adjusting the airway, and, if necessary, removing secretions [2]. The baby was immediately placed on a radiant warmer, and secretions from the mouth and nose were removed using a bulb syringe. The airway was opened by performing a jaw-thrust procedure (Table 1).

**Table 1:** NRP guidelines for newborn resuscitation. NRP: Neonatal Resuscitation Program.

Step	Action
1. Warmth	Place the infant on a radiant warmer
2. Airway	Adjust airway and remove secretions if necessary
3. Breathing	Start supplementary oxygen if SpO2 remains below 90%
4. Circulation	Begin chest compressions if heart rate is below 60 bpm
5. Intubation	Perform intubation if required
6. Medication	Administer epinephrine if necessary

Oxygen therapy

As long as the baby's SpO2 remained below 90%, supplementary oxygen was started using a flow-inflating bag, mask, and mixed oxygen/air combination [3]. The baby's SpO2 gradually increased until it reached 92%.

Chest compressions

The baby's heart rate continued to be below 60 beats per minute, necessitating the start of chest compressions in accordance with NRP recommendations [4]. With the two-thumb encircling approach, a 3:1 compression-to-ventilation ratio was used. The infant's heart rate reached 100 beats per minute after two minutes of chest compressions.

Intubation and medication administration

Endotracheal intubation was carried out with a 3.0-mm uncuffed endotracheal tube due to the patient's continued respiratory distress and the requirement for continuous positive-pressure ventilation [5]. Following that, the infant received epinephrine (0.01 mg/kg) via the endotracheal tube in accordance with NRP recommendations [6].

Post-resuscitation management

**Figure 1:**
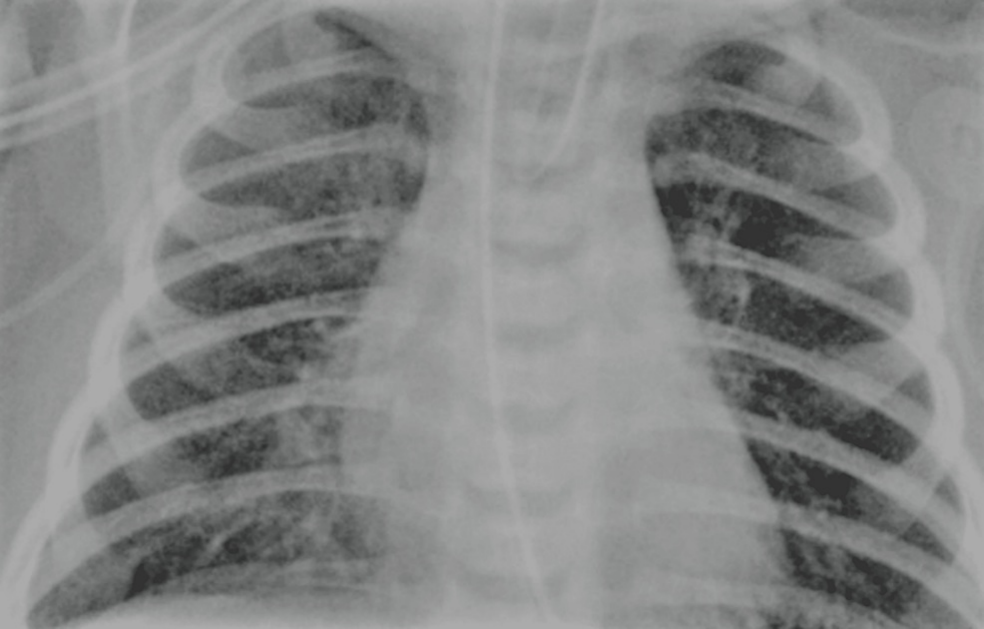
Chest X-ray showing pneumonia.

The newborn was taken to the neonatal intensive care unit (NICU) for additional therapy after resuscitation. Broad-spectrum antibiotics, including ampicillin and gentamicin, were started after a chest X-ray (Figure 1), which revealed diffuse bilateral infiltrates suggestive of pneumonia [7, 8]. Despite administering antibiotics, blood cultures and viral testing yielded negative results. The baby's condition steadily improved, and he was extubated on the fifth day of his hospital stay. On day 14, he was sent home with no obvious neurological aftereffects (Table 2).

**Table 2:** Timeline of infant's hospital stay. NICU: Neonatal intensive care unit.

Day	Event
1	Infant brought to ER, resuscitation performed, admitted to NICU
2-4	Infant treated with broad-spectrum antibiotics for pneumonia
5	Infant extubated, gradual improvement in condition
6-13	Continued monitoring and supportive care
14	Infant discharged home with no apparent neurological deficits

## Discussion

This case report describes the successful NRP-recommended ER resuscitation of a two-month-old baby [9]. It is important to note that the patient in this case presentation is a full-term infant who was two months old at the time of presentation. Early detection of respiratory distress and prompt application of resuscitation techniques were essential to this two-month-old infant's successful recovery. The NRP guidelines offer a methodical approach, ensuring that medical professionals are equipped to handle such cases successfully [10]. In this instance, the two-month-old baby had respiratory distress and cyanosis, which called for quick action. The NRP recommendations strongly emphasize the significance of the initial measures, which include providing warmth, establishing the airway, and, if necessary, cleaning secretions [11]. These actions were swiftly carried out, enabling the start of oxygen therapy and necessary chest compressions. It has been demonstrated that using a blended oxygen/air combination reduces the risk of oxidative stress and improves outcomes for preterm newborns [12]. In this instance, the two-month-old infant's SpO2 levels were effectively raised by the blended oxygen/air mixture. The two-month-old baby's heart rate quickly improved when chest compressions were started in accordance with the NRP recommendations [13]. Given the infant's ongoing respiratory distress, endotracheal intubation and drug administration in accordance with NRP standards were crucial in this situation [14]. The NRP's protocols were followed when administering epinephrine via the endotracheal tube, which helped to stabilize the two-month-old infant [15].

The excellent outcome in this instance was attributed to the early identification of respiratory distress, adherence to NRP recommendations, and adequate post-resuscitation care, including the use of broad-spectrum antibiotics. This demonstrates how crucial it is for healthcare workers involved in newborn care to get adequate training and follow the NRP recommendations [16, 17]. While the successful resuscitation of a two-month-old baby according to NRP guidelines is described in this case report, it is crucial to note that this is only one case study and that general conclusions cannot be drawn from it. To determine the usefulness of NRP in this age group, additional research is required, including comparisons with other resuscitation algorithms. It is crucial to assess the results of these various approaches in this particular age group because, depending on the healthcare facilities, both Pediatric Advanced Life Support (PALS) and NRP may be provided to newborns in this age range. This instance emphasizes the relevance of early detection of respiratory distress and the fast implementation of effective resuscitation procedures, underscoring the significance of healthcare providers having sufficient training in newborn resuscitation to improve outcomes.

## Conclusions

In this case report, a two-month-old baby is successfully revived in the ER, highlighting the crucial need to follow the NRP recommendations for effective results. The NRP standards' comprehensive approach guarantees medical professionals have the necessary tools to properly handle newborn crises. Improved outcomes for neonates who require resuscitation depend on thorough training and rigorous adherence to the NRP recommendations.
